# Personality functioning and mental distress in leaders of small- and medium sized enterprises

**DOI:** 10.1371/journal.pone.0312675

**Published:** 2024-11-20

**Authors:** Rebecca Erschens, Carla Schröpel, Sophia H. Adam, Harald Gündel, Peter Angerer, Ulrike Dinger-Ehrenthal, Johannes C. Ehrenthal, Mathias Diebig, Sophie Hofmann, Michael Gast, Susan Gritzka, Stephan Zipfel, Florian Junne

**Affiliations:** 1 Department of Psychosomatic Medicine and Psychotherapy, University Hospital Tuebingen, University of Tuebingen, Tuebingen, Baden-Wuerttemberg, Germany; 2 Department of Psychosomatic Medicine and Psychotherapy, Ulm University Medical Center, Ulm, Germany; 3 Institute of Occupational, Social and Environmental Medicine, Centre for Health and Society, Faculty of Medicine, Heinrich-Heine-University Düsseldorf, Düsseldorf, Nordrhein-Westfalen, Germany; 4 Department of Psychosomatic Medicine and Psychotherapy, Medical Faculty of the Heinrich Heine University Düsseldorf, Düsseldorf, Nordrhein-Westfalen, Germany; 5 Department of Psychology, University of Cologne, Cologne, Germany; 6 Work and Organizational Psychology, Trier University, Trier, Germany; 7 Department of Psychosomatic Medicine and Psychotherapy, Otto von Guericke University Magdeburg, University Hospital Magdeburg, Magdeburg, Germany; Medical University of Vienna, AUSTRIA

## Abstract

**Objective:**

For leaders of small and medium-sized enterprises (SMEs) grappling with diverse tasks and stressors, personality functioning can play a key role on stress perception and building and managing professional relationships. We investigated the relationship between SME leaders’ personality functioning on the dimensions of self-perception and self-regulation on perceived stress reactivity (PSR), and symptoms of anxiety and depression.

**Methods:**

A cross-sectional sub analysis of a multi-centre RCT project was performed. A sample of *N* = 174 SME leaders from various sectors in southern Germany were examined on their self-rated levels of personality functioning (OPD-SQ), involving self-perception (facets: self-reflection, affect differentiation, sense of identity) and self-reflection (facets: impulse control, affect tolerance, regulation of self-esteem), with higher scores indicating lower levels of personality functioning. The outcome variables were perceived stress reactivity (PSRS) and symptoms of depression and anxiety (HADS).

**Results:**

The mean score for symptoms of anxiety was *M* = 6.13 (*SD* = 3.34), depression *M* = 4.40 (*SD* = 3.35), and for PSRS *M* = 21.39 (*SD* = 8.04). The mean sum score for self-perception was *M* = 7.71 (*SD* = 7.19) and for self-regulation *M* = 10.50 (*SD* = 7.09). The results of three regression models showed that higher scores for affect differentiation (*r*_*sp*_ = .13), impulse control (*r*_*sp*_ = .14) and regulation of self-esteem (*r*_*sp*_ = .29) were associated with higher scores for PSR. Higher scores for affect differentiation (*r*_*sp*_ = .17) and affect tolerance (*r*_*sp*_ = .20) were significantly associated with higher scores for anxiety. A higher score for regulation of self-esteem (*r*_*sp*_ = .17) was associated with higher depression scores.

**Conclusion:**

This study highlights the association between core dimensions of SME leaders’ personality functioning and mental distress. The findings can be applied on interventions and health promotion and the establishment of high-quality professional relationships and leadership skills.

## Introduction

Leaders of small and medium-sized enterprises (SMEs) are confronted with versatile specific stressors, such as holding multiple roles, working in sandwich or sole decision-making positions [1, 2]. Also, SME leaders often have limited opportunities to communicate about how to cope with their demanding roles [1]. These circumstances are frequently associated with distress and detrimental health outcomes and the reduction of favourable leadership traits which might in turn negatively affect the entire team. In turn, building high-quality professional relationships and managing relational and health-oriented leadership plays a significant role in shaping the work environment on SMEs affecting the whole team’s health and well-being [3, 4]. However, there are few interventions to promote mental health and foster stress reduction for SME leaders [5].

The intrapersonal ability to perceive and regulate one’s own emotional states plays an essential role in the development of stress and stress-related health outcomes. Therefore, constructs regarding self-regulation and self-perception are of particular interest for SME leaders’ behavioural patterns and their experience of stress and subsequent stress sequelae. These variables can be conceptualized as part of personality functioning, within the Operationalized Psychodynamic Diagnosis system (OPD, [6, 7]) which has recently been incorporated as a dimensional approach to the *Alternative Model of Personality Disorders* in DSM-5 [8] and the ICD-11 [9]. For this purpose, self-related scales of the Levels of Structural Integration Axis (LSIA; i.e. *self-perception* and *self-regulation*) including the corresponding subscales (see Material section for a detailed description) were chosen for this research.

The concept of personality functioning has so far mostly been used and researched within clinical settings and rarely in the field of leadership and health promotion. However, the underlying mechanisms can be equally transferable and relevant to the SME leadership context as leaders in SMEs often have to manage team dynamics and balance the projections of their followers in order to ensure stability [10]. Therefore, an increased awareness and knowledge of biographical reappraisal and personality functioning can be beneficial for the individual and collective well-being within the SME: Knowledge of personality functioning abilities and vulnerabilities can assist leaders in distancing themselves from current impulses, emotions and self-evaluations, enabling them to implement alternative modes of experience and regulation and enhancing their self-regulatory competence. For example, interpersonal emotion regulation was found to be associated with team effectiveness and innovation [11] and reduced self-regulatory mechanisms in experiencing external stressors were found to be a major risk factor for burnout at work [12]. Also, previous studies have indicated that leaders with good health tend to exhibit more positive and transformational behaviour, which then leads to the improvement of their own well-being and that of their followers [13–17]. Also, levels of personality functioning can affect leadership styles, the general mood and atmosphere in the team and may thus promote or hinder the individual career opportunities of the followers [18]. In addition to the research-aspect, there is growing interest and demand for coaching for leaders regarding self-regulatory capacities [19].

We thus assume that personality functioning (i.e. *self-perception* and *self-regulation*) may serve as an antecedent regarding both, subclinical outcomes operationalized by perceived stress reactivity (PSR) and clinically relevant mental health outcomes represented by depression and anxiety symptoms. The concept of PSR will be further addressed using the concept by Schlotz et al. [20]. PSR comprises individual physical and psychological responses to stressors that, in turn contribute to the occurrence of distress and associated disorders. The concept is founded upon the transactional model of stress and coping, which asserts that stress is not solely a function of external events but is also contingent upon how these events are appraised and responded to by the individual. PSR, therefore, constitutes an integral aspect of this appraisal process, influencing both the experience of stress and the outcomes of stress exposure. Limm et al. [21] found that elevated levels of PSR correlated with negative health outcomes such as depression, anxiety, chronic stress, and health-related complaints in middle-aged employees. Also, personality functioning was found to be related to heightened levels of perceived distress [22].

**Fig 1:**
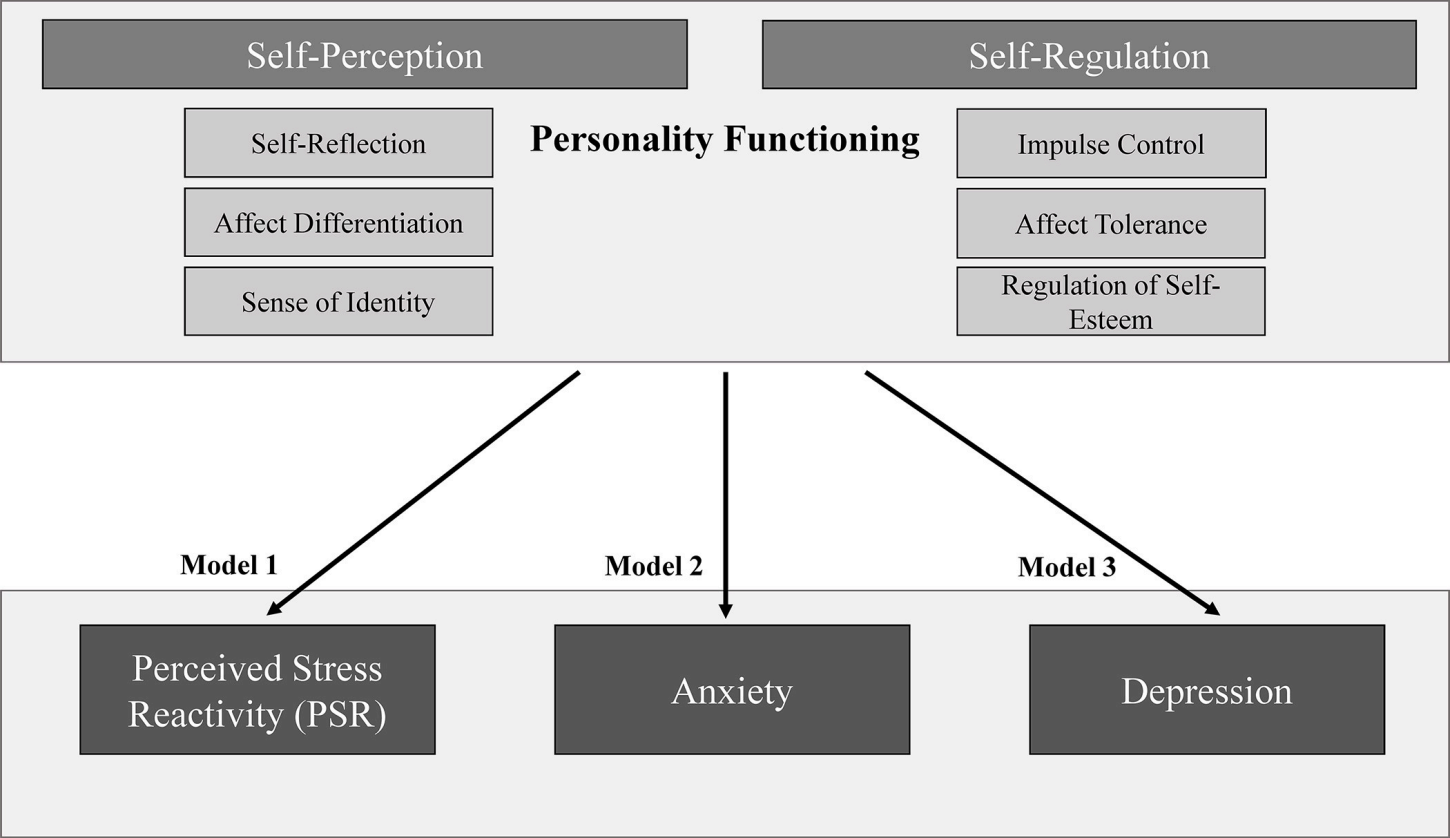
Illustration of the underlying conceptual model of the study exploring the relationship between SME leaders’ personality functioning on the dimensions of self-perception and self-regulation and perceived stress reactivity and symptoms of anxiety and depression.

Thus, the overarching aim of this study is to investigate the contribution of personality functioning (operationalized by the subscales of *self-perception* and *self-regulation*) on mental distress (operationalized by PSR and symptoms of anxiety and depression) among SME leaders. Fig 1 presents the conceptual model that illustrates the assumed influence of self-rated personality functioning on the manifestation of perceived stress reactivity and symptoms of depression and anxiety. Hence, the model integrates SME leaders’ personality functioning in the dimensions of self-perception, self-regulation, stress reactivity and symptoms of anxiety and depression.

More specifically, we hypothesise that low levels of SME leaders’ personality functioning in the dimensions of self-perception and self-regulation are associated with higher levels of PSR (Model 1), anxiety (Model 2) and depression (Model 3). To the best of our knowledge we are not aware of any other study that has examined the association between facets of personality functioning and PSR, depression and anxiety and among leaders in SMEs.

## Methods

### Sample and procedure

The present study is a baseline-analysis of a multi-centre randomised control trial [2]. The data collection period was May to August 2021. Leaders were recruited through email, telephone, and via information sessions. To qualify for the study, leaders must be working in an SME (<500 employees), have leadership responsibility for at least on one follower and be aged between 18 to 65, with no intention of retiring within the next year. The leaders provided informed consent online before proceeding to complete the online survey [23].

### Material

The questionnaire was comprised of questions concerning demographic data, inquiries on health behaviour, and validated questionnaires gauging health-related concepts. In the following, the questionnaires used are described and values for internal consistency from the manuals of the respective questionnaires and scales are reported.

#### Operationalized Psychodynamic Structure Questionnaire (OPD-SQ)

The Operationalized Psychodynamic Structure Questionnaire (OPD-SQ, [24]) was used to assess personality functioning. For the present study we used two subscales consisting of three facets each: **self-perception** (12 items, facets: *self-reflection*, *affect differentiation*, *sense of identity*) and **self-regulation** (13 items, facets: *impulse control*, *affect tolerance*, *regulation of self-esteem*; for a detailed overview see [25]). Each facet consists of four items, except for affect tolerance, which consists of five items. Ratings are given on a scale from 0 (no agreement at all) to 4 (very high agreement). Higher values indicate a higher level of self-reported structural impairment according to the terminology of the OPD, representing lower levels of personality functioning. Cronbach’s alpha for internal consistency ranges from α = .88 and .91 for self-perception and between α = .82 and .84 for self-regulation.

#### Perceived Stress Reactivity (PSRS)

The Perceived Stress-Reactivity Scale (PSRS; [26]) contains 23 items gauging stress reactivity, with individual response options ranging from 0–2. In addition to the total score (range 0–46), five subscale sum scores are derivable: *prolonged reactivity*, *reaction to failure*, *reactivity to social conflicts*, *reacting to work overload* and *reacting to social evaluation*. A higher mean score indicates a higher level of stress reactivity. Cronbach’s alpha for the total score is α = .89.

#### Anxiety and Depression (HADS)

The Hospital Anxiety and Depression Scale-German version (HADS-D; [27]) was used. The questionnaire consists of the subscales *anxiety* and *depression* and comprises 14 items (7 per subscale) that are rated using a 4-point scale with item-specific response options ranging from 0 to 3. Sum scores for the subscales can be calculated between 0–21, with scores up to 7 denoting a normal symptom load and a score between 8 and 10 indicating a mild symptom burden. A score between 11 to 14 indicates moderate symptoms of depression or anxiety, while 15 or more suggests severe symptoms [28]. The measure of Cronbach’s alpha for depression is α = .81 and for anxiety it is α = .80.

### Statistical analysis

We used SPSS version 28.0 (IBM, Armonk, NY, USA) to perform statistical analysis. The level of significance was set at α = .05. In addition to the descriptive analysis, we calculated Pearson correlation coefficients and Cronbach’s alpha for all scales, subscales and facets included in the study. To analyse if low levels of SME leaders’ personality functioning in the dimensions of self-perception and self-regulation are associated with higher levels of perceived stress reactivity (PSR) and symptoms of anxiety and depression we conducted three separate linear regression models. The dependent variables were perceived stress reactivity (PSRS, Model 1), anxiety (HADS, Model 2) and depression (HADS, Model 3). For each model we entered the facets *self-reflection*, *affect differentiation* and *sense of identity* (self-perception subscale, OPD-SQ) and the *facets impulse control*, *affect tolerance* and *regulation of self-esteem* (self-regulation subscale, OPD-SQ) as independent variables. For effect sizes, we examined semi-partial correlations as the unique relation between a dependent and the independent variable, with *r*_*sp*_ = .10 indicating a small effect, *r*_*sp*_ = .30 indicating a medium effect, and *r*_*sp*_ = .50 indicating a large effect, see Cohen [29]. Please note, that in order to maintain consistency with the other questionnaires and scales used in our study, we used the sum score as the basis for calculating the means of the scales and facets of the OPD-SQ. Hence, to compare the mean values of this study with the mean values of reference samples in the discussion, we divided our mean values by the number of items of each facet or each scale.

## Results

### Response rate and sample description

*N* = 174 (RR = 77.3%) leaders took part in the study. The majority of participants were male (*n* = 124, 71.3%). Leader´s age ranged between 26 and 61 years (*M* = 45.51, *SD* = 8.98). The majority of leaders held a university degree (Master/Diploma: *n* = 88, 51.2%, Bachelor: *n* = 25, 14.5%) or a technical college degree (*n* = 25, 14.5%). Overall, *n* = 17 (9.9%) had completed a professional training and *n* = 13 (7.6%) held a PhD degree, while *n* = 4 (2.3%) did not indicate to have a vocational qualification. Leaders were employed across diverse sectors, the majority held jobs in finance, insurance, law, taxation, IT, telecommunications, manufacturing, crafts, or research and development. The majority of leaders indicated having either no children (*n* = 66, 37.9%), two children (*n* = 54, 31.0%), or only one child (*n* = 30, 17.2%). In total *n* = 24 (13.8%) reported having three or more children and the majority (*n* = 153, 87.9%) reported being in a relationship.

### Descriptive statistics of the analysed variables

Table 1 provides a psychometric overview for all variables used in the study. The mean sum score for self-perception was *M* = 7.71 (*SD* = 7.19) and for self-regulation *M* = 10.50 (*SD* = 7.09). The mean score for PSRS was *M* = 21.39 (*SD* = 8.04). On the PSRS subscales, SME-leaders reported a mean score of *M* = 3.41 (*SD* = 2.06) for prolonged reactivity, *M* = 3.54 (*SD* = 2.28) for reactivity to work overload, *M* = 6.37 (*SD* = 2.27) for reactivity to social conflicts, *M* = 4.59 (*SD* = 1.65) for reactivity to failure and *M* = 3.47 (*SD* = 2.39) for reactivity to social evaluation. Overall, SME leaders showed a mean score of *M* = 6.13 (*SD* = 3.34) for anxiety and *M* = 4.40 (*SD* = 3.35) for depression. The majority of participants displayed no to low symptoms of anxiety (66.1%) and depression (81.6%). For an overview of the percentage distribution on the HADS see Table 2.

**Table 1:** Table of means of the study variables for the total sample (*N* = 174).

	scale range^1^	*M*	*SD*	min	max
**OPD-SQ**					
**self-perception**					
scale sum score	(0–48)	7.71	7.19	0	41
self-reflection	(0–16)	2.90	2.63	0	12
affect differentiation	(0–16)	3.10	3.31	0	16
sense of identity	(0–16)	1.70	2.38	0	13
**self-regulation**					
scale sum score	(0–52)	10.50	7.09	0	42
impulse control	(0–16)	4.40	3.01	0	16
affect tolerance	(0–20)	2.29	3.20	0	17
regulation of self-esteem	(0–16)	3.77	2.56	0	13
**PSRS**					
scale sum score	(0–46)	21.39	8.04	5	40
prolonged reactivity	(0–8)	3.41	2.06	0	8
reactivity to work overload	(0–10)	3.54	2.28	0	9
reactivity to social conflicts	(0–10)	6.37	2.27	1	10
reactivity to failure	(0–8)	4.59	1.65	0	8
reactivity to social evaluation	(0–10)	3.47	2.39	0	10
**HADS**					
scale sum score	(0–42)	10.53	5.90	1	29
anxiety	(0–21)	6.13	3.34	0	15
depression	(0–21)	4.40	3.35	0	15

^1^The minimum and maximum scores that can be achieved on each subscale are given in brackets.

**Table 2:** Percentages for symptoms of anxiety and depression on the HADS categorized by symptom severity.

score	symptom severity	anxiety	depression
**0–7**	**normal**	66.1%	81.6%
**8–10**	**mild**	23.6%	12.6%
**11–14**	**moderate**	9.2%	4.6%
**15–21**	**severe**	1.1%	1.1%

All study variables were significantly correlated with each other (*r* = .22 to *r* = .91) and the majority of the effect sizes of the correlations were moderate or strong. See S1 Table in the supplements for an overview of the intercorrelations and internal consistencies for all scales and subscales. Cronbach’s alpha ranged from α = .67 (acceptable) to α = .91 (excellent).

### Results of the linear regression analyses

We tested the assumptions for conducting linear regression models. Graphical analyses revealed possible heteroskedasticity in our data for Model 1 and Model 2. Therefore, we decided to use a heteroskedasticity-consistent standard error estimator (HCSE) to reduce the effects of heteroskedasticity. In line with recommendations from literature, we chose the HC3 estimator [30, 31]. Further graphical analysis revealed a violation of the normality assumption for Model 3, so we decided to provide additional bootstrap confidence intervals to prove significance of coefficients (95% bootstrap bias-corrected and accelerated confidence intervals, 1000 samples). All other assumptions were fulfilled.

**Fig 2:**
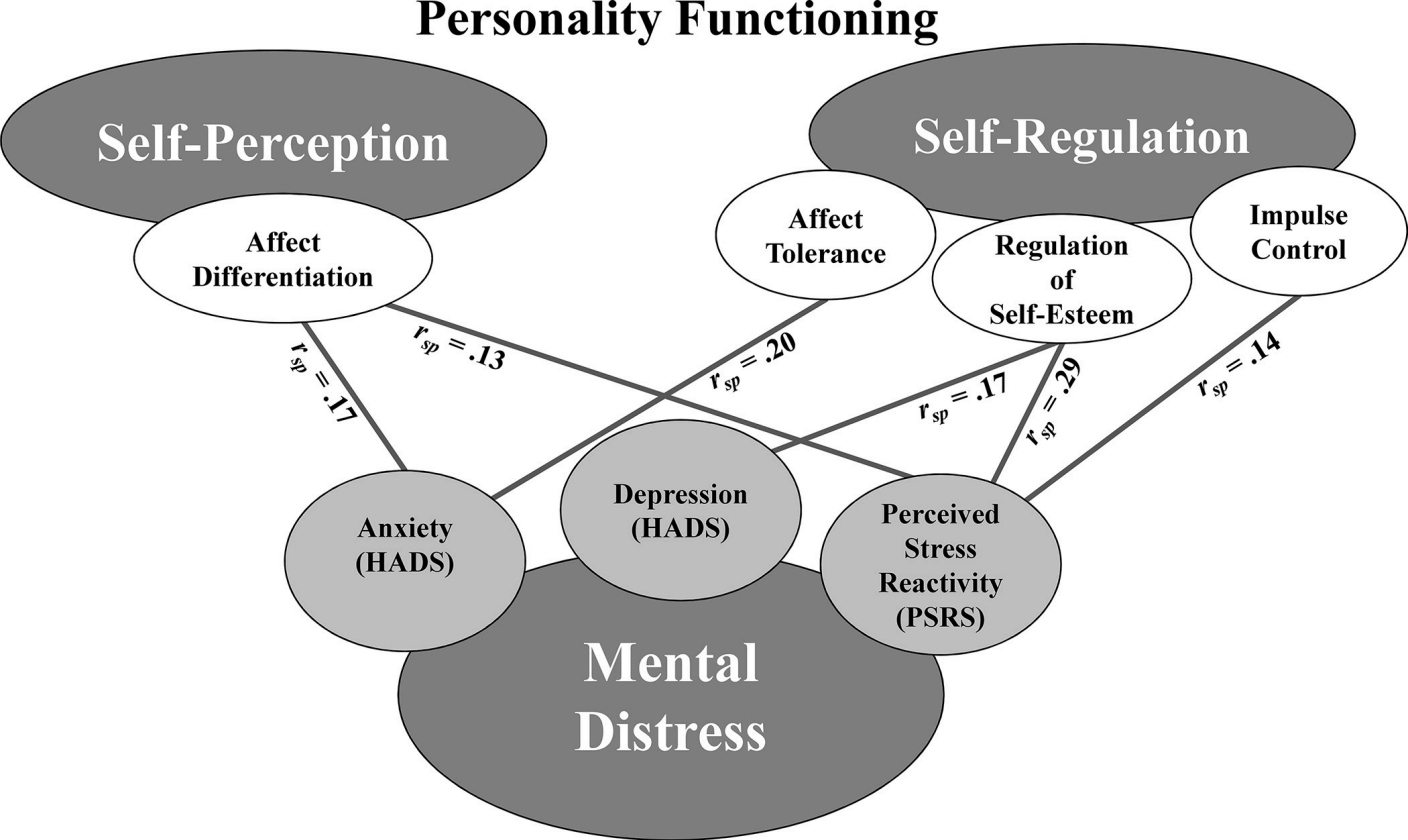
Illustration of the aggregated results model showing significant associations between SME leaders’ personality functioning (on the core facets of affect differentiation, affect tolerance, regulation of self-esteem and impulse control) and mental distress (perceived stress reactivity and symptoms of anxiety and depression). ***Note*.** semi-partial correlation coefficients *r*_*sp*_ indicating the unique relation of each independent variable with the corresponding dependant variable.

Model 1 predicting perceived stress reactivity (PSR) was significant with *F*(6, 167) = 33.18, *p* < .001. Higher scores affect differentiation (*r*_*sp*_ = .13), impulse control (*r*_*sp*_ = .14) and regulation of self-esteem (*r*_*sp*_ = .29) were associated with higher scores for PSR. No significant associations emerged for the other predictor variables. Model 2 predicting anxiety was significant with *F*(6, 167) = 22.45, *p* < .001. Higher scores for affect differentiation (*r*_*sp*_ = .17) and affect tolerance (*r*_*sp*_ = .20) were associated with higher anxiety scores. No significant associations emerged for self-reflection, sense of identity, impulse control and regulation of self-esteem. Model 3 predicting depression was significant with *F*(6, 167) = 10.61, *p* < .001. Higher scores for regulation of self-esteem (*r*_*sp*_ = .17) were associated with higher depression scores. This result could be confirmed by the bootstrap interval, B = 0.29 [0.03; 0.54]. There were no significant associations with the other predictors. All significant *p*-values would have survived Bonferroni-correction for multiple testing. All models showed a high goodness-of-fit [29]. See Table 3 for an overview on all regression coefficients, test statistics and effect sizes. For a graphical illustration of the significant results, see Fig 2.

**Table 3:** Results of the three regression analyses for PSR (Model 1), anxiety (Model 2) and depression (Model 3).

	Model 1: perceived stress reactivity (PSR)						Model 2: anxiety						Model 3: depression					
	*N* = 174, adjusted *R*^2^ = .53						*N* = 174, adjusted *R*^2^ = .43						*N* = 174, adjusted *R*^2^ = .25					
	^**1**^ **B**	^**2**^ **SE(B)**	^**2**^ **t**	^**2**^ **p**	**95%CI(B)**	^3^ **r**_***sp***_	^**1**^ **B**	**SE(B)**	**t**	**p**	**95%CI(B)**	**r**_***sp***_	^**1**^ **B**	^**2**^ **SE(B)**	^**2**^ **t**	^**2**^ **p**	**95%CI(B)**	**r**_***sp***_
**self-perception**																		
self-reflection	0.14	0.19	0.72	.473	[-0.30; 0.58]	.03	0.03	0.10	0.31	.754	[-0.17;0.23]	.02	-0.02	0.12	-0.17	.867	[-0.25; 0.21]	-.01
affect differentiation	0.55	0.18	3.02	.003	[0.11; 0.99]	.13	0.29	0.12	2.55	.012	[0.09;0.49]	.17	0.23	0.12	1.97	.051	[0.00; 0.46]	.13
sense of identity	0.02	0.26	0.09	.931	[-0.47; 0.52]	< .01	0.03	0.12	0.22	.825	[-0.20; 0.25]	.01	0.20	0.13	1.50	.136	[-0.06; 0.46]	.10
**self-regulation**																		
impulse control	0.44	0.17	2.63	.009	[0.12; 0.76]	.14	0.03	0.07	0.46	.646	[-0.11; 0.18]	.03	0.13	0.08	1.51	.134	[-0.04; 0.30]	.10
affect tolerance	0.20	0.21	0.98	.330	[-0.22; 0.63]	.05	0.35	0.10	3.53	< .001	[0.15; 0.54]	.20	-0.04	0.11	-0.33	.745	[-0.26; 0.19]	-.02
regulation of self-esteem	1.23	0.21	5.96	< .001	[0.80; 1.66]	.29	0.09	0.11	0.78	.436	[-0.11; 0.28]	.05	0.29	0.11	2.56	.012	[0.07; 0.52]	.17
intercept	12.21	0.90	13.57	< .001	[10.44; 13.97]		3.81	0.43	8.94	< .001	[3.00; 4.62]		1.83	0.47	3.90	< .001	[0.90; 2.75]	

^1^unstandardised coefficient

^2^recalculation of *SE*, *t* and *p* using heteroskedasticity-consistent standard error estimator HC3

^3^semi-partial correlation coefficients

*Note*. significant results are highlighted in grey

## Discussion

The study presented herein was conducted to analyse leaders of small-and medium sized enterprises (SME) personality functioning in the dimensions of self-perception (facets: *self-reflection*, *affect differentiation*, *sense of identity*) and self-regulation (facets: *impulse control*, *affect tolerance*, *regulation of self-esteem*) with concerning perceived stress reactivity (PSR) and symptoms of anxiety and depression. For further interpretation, it should be noted that higher scores on the subscales of the Operationalized Psychodynamic Structure Questionnaire (OPD-SQ) indicate lower levels of personality functioning.

The findings of the study are presented below and discussed in the context of existing literature. Subsequently, perspectives on the impact on the mental health of leaders, the leader-member dynamic and interventions are considered, followed by a discussion of the methodological limitations.

### Stress, anxiety and depression among leaders

Our results indicate a mean total score of *M* = 21.39 on the perceived stress reactivity scale (PSRS) which is comparable to the PSRS mean score for a German reference sample by Schlotz et al. [26] of individuals aged 26 to 60 (*M* = 20.43–25.23). Schlotz et al. reported their reference values separately for men and women rather than as a combined value. Thus, in brackets, the mean is given first for men and then for women. Further, the same reference sample reported comparable mean scores for the PSRS subscales for reactivity to work overload (reference *M* = 3.56–4.70 vs. our sample *M* = 3.54), for reactivity to social conflicts (reference *M* = 5.59–7.13 vs. our sample *M* = 6.37) and for reactivity to failure (reference *M* = 4.54–4.93 vs. our sample *M* = 4.59). In a descriptive comparison, the leaders in our sample had slightly higher scores for prolonged reactivity (reference *M* = 2.85–3.34 vs. our sample *M* = 3.41) and slightly lower scores for reactivity to social evaluation (reference *M* = 3.82–5.26 vs. our sample *M* = 3.47; refer to Schlotz et al. [26]). In our sample, overall 22.3% of all SME leaders showed slightly elevated scores (8 or greater) for anxiety and 12.6% elevated scores for depression. Further the mean value for anxiety was *M* = 6.13 and for depression *M* = 4.40, which corresponds to findings by Afonso et al. [32] using a comparable non-clinical working population. Compared to a non-clinical general German reference sample [33], SME leaders in our study showed a higher percentage of elevated scores (8 or greater) for anxiety (reference sample: 21.0%) and a lower percentage of elevated depression scores (reference sample: 23.7%).

### Personality functioning and mental distress

SME leaders assessed their own personality functioning in terms of self-perception and self-regulation as being at a relatively high structural level when compared to a non-clinical reference sample by Ehrenthal and colleagues [24]. In this reference sample, the mean value for self-perception was *M* = 0.89 (vs. our sample *M* = 0.64) and for self-regulation *M* = 1.15 (vs. our sample *M* = 0.81). In the same non-clinical reference sample the mean score for the facet self-reflection was *M* = 0.94 (vs. our sample *M* = 0.73), for affect differentiation it was *M* = 1.02 (vs. our sample *M* = 0.78), for sense of identity it was *M* = 0.72 (vs. our sample *M* = 0.43), for impulse control it was *M* = 1.12 (vs. our sample *M* = 1.10), for affect tolerance it was *M* = 0.86 (vs. our sample *M* = 0.46), and for regulation of self-esteem it was *M* = 1.37 (vs. our sample *M* = 0.94). Please note that to compare our mean values to the reference sample by Ehrenthal and colleagues [24], we have divided our mean values reported in the results section by the number of items of each facet or scale (see statistical analysis, method section).

Given that the majority of research on personality functioning was performed on clinical or convenience samples [34–37], our findings can be incorporated and discussed within the existing literature only to a limited extent. However, the data provide valuable insights for the specific population of SME leaders which is in line with recent aspirations to foster leaders’ regular reflection on their behaviour and decision-making processes due to their considerable responsibility [38]. Thus, the results of our study suggest that specific intrapersonal factors among SME leaders are associated with an increased or decreased risk of experiencing mental distress.

Our results indicate that one facet of the subscale self-perception (i.e. affect differentiation) and three facets of the subscale self-regulation (i.e. affect tolerance, regulation of self-esteem and impulse control) substantially contribute to the explanation of mental distress among SME leaders. These facets thus may represent relevant *core* dimensions of personality functioning on SME leaders’ mental health. The following sections will discuss these core dimensions and their relevance for both the intrapersonal aspects of leaders and the interpersonal level in leader-follower and leader-team dynamics.

Firstly, we found significant positive associations between the facet *affect differentiation* (*I often have feelings that I can´t understand; There is often such a chaos of feelings inside me that I can’t even describe it*) and symptoms of anxiety and PSR. Affect differentiation is the capacity to recognize, comprehend a variety of one’s own emotional states, embracing their full spectrum of nuances (see the OPD-3 manual [39]). Impairment of this ability to read internal affective states in leaders is often accompanied by difficulties in identifying, organizing, and adequately regulating one’s own feelings, which could result in a sense of emotional disorganisation, leading to stress and anxiety. Given that leaders frequently have to make decisions under pressure, a lack of awareness and expression of emotional reactions can emotional instability and, consequently, a reduction in the quality of leadership and inadequate or even destructive leadership behaviour [16, 40].

Moreover, we found a significant positive association between the facet *affect tolerance* (*Sometimes my feelings are so intense that I get scared; Sometimes the only thing I feel is panic*) and symptoms of anxiety. Affect tolerance is defined as the capacity to endure and manage intense emotional experiences without being overwhelmed by their effects. A deficiency in affect tolerance is characterised by an inability to cope with intense emotions, particularly those of a negative nature such as fear, anger or sadness [39] and can result in experiencing intense emotions to a degree that they may find overwhelming. A leader with limited affect tolerance may be more prone to reaching their emotional limits in stressful situations. Also, they might be more likely to employ coping strategies such as emotional suppression in order to self- regulate their emotions which in turn lead to impaired leadership outcomes [41]. This can result in a depletion of the resources available for subsequent interactions [42]. In contrast to appropriate emotion regulation, these leaders may tend to overreact due to emotional exhaustion, withdraw emotionally or react with fear or panic in crisis situations. This can subsequently lead to obstructive micromanagement and a reduction in the exchange between leader and member (see for an overview [43]), which can unsettle employees and have a negative impact on the overall working atmosphere at a higher level [1].

Furthermore, we found significant positive associations between the facet *regulation of self-esteem* (*I don’t have good self-esteem; I often feel useless and dispensable)* and symptoms of depression and PSR. Regulation of self-esteem describes an individual’s capacity to maintain a stable and positive self-image in everyday as well as stressful or critical circumstances. An inability to regulate one’s self-esteem can result in the development of low self-esteem and a negative self-perception. Consequently, those in managerial roles may perceive themselves as inferior, useless, or replaceable [39]. The experience of being undervalued or the perception of lacking competence can result in a decline in self-esteem and doubts about one’s own abilities and self-worth and low self-efficacy (for an overview see [44, 45]) which may in turn contribute to the onset of symptoms of depression. In a same vein, the findings of this study indicate that limited self-esteem regulation in leaders is associated with an increased risk of developing stress and depression, as a persistently negative self-image may emerge. However, a stable sense of self-esteem is of particular importance in leadership roles, as leaders are frequently subjected to criticism and scrutiny in their decision-making processes and their leadership behavior [46]. An unstable sense of self-esteem can cause to react more severely to failures or criticism, thereby increasing the risk of developing depressive symptoms. Thus, the in line with previous findings by Perlinger et al. [12], leaders’ capacity to successfully self-regulate can therefore serve as a protective factor for the leaders themselves. Hence, a leader who experiences difficulties at managing their own self-esteem may be perceived by their followers as hesitant or insecure, which can have deleterious effects on the working atmosphere and motivation within the whole team. This is particularly pertinent in the context of the inherently dynamic working environment characteristic of SMEs, where interpersonal conflicts have the potential to rapidly impact the entire team [47].

Lastly, we found a significant positive association between the facet *impulse control* (*Sometimes I’m so full of rage that I can’t guarantee anything; Sometimes I explode like a powder keg*) and PSR. Impulse control refers to the capacity to regulate and suppress intense emotional impulses, such as anger or irritation, in everyday as well as challenging circumstances. Impaired impulse control indicates that an individual encounters difficulty in controlling strong impulses, which can result in impulsive and inappropriate reactions [39]. Hence, deficits on this core dimension on SME leaders might be associated with the reduced ability to experience, suspend or inhibit (aggressive) impulses reduces their perceptions of perceived stress. Previous research by Tafvelin et al. [48] has demonstrated an association between leaders’ personality traits with leadership behavior. More specifically, neuroticism in leaders was found to be positively associated with passive and active destructive leadership and work stress. Thus, as reflected in our results regarding the facet impulse control, leaders might be unable to respond promptly and rationally during conflicts, as they should, but instead tend to exhibit aggressive outbursts due to depleted resources. Thus, SME leaders who exhibit poor impulse control can be more prone to experiencing elevated PSR as it makes them more likely to perceive situations as stressful and to respond emotionally. A cyclical process may form whereby stressors give rise to emotional outbursts, which in turn serve to intensify perceived distress. In addition, a leader that is perceived as erratic or emotionally unstable can erode employees’ confidence in their leadership abilities, which has a detrimental effect on the working environment. This is in line with the general notion that an increased level of distress is associated with more negativity experienced in relationships [49]. Hence, frequent emotional, often aggressive outbursts have the potential to erode the trust and respect between leader and followers, leading to diminished communication, reduced collaboration, and lower levels of employee engagement in the long-term. Consequently, their capacity to serve as a role model, which is a fundamental aspect of health-related leadership–e.g. transformational leadership, is consequently diminished [50, 51].

As mentioned earlier, owing to the genesis of OPD-SQ, there is still limited exploration on personality functionality through the self-report mechanism among non-clinical populations. However, Dinger et al. [34] have shown substantial correlations between self-ratings using the OPD-SQ and observer-rated clinical interviews, supporting the notion that personality functioning can be validly assessed using self-report measures in non-clinical populations. Considering two possible ways to interpret the OPD-SQ scale, both aspects should be considered: in line with previous research by Dinger et al. [34] and Ehrenthal et al. [24], non-clinical participants scored lower on the self-rating, indicating higher levels of structural integration. Although a bias in this direction is rather unlikely given the highly structured population studied in this study, the results can be interpreted in the sense that low scores indicate low insight into individual deficits, which in turn is more of a risk factor in terms of a blind spot in self-perception and thus the development of intra- and interpersonal problems (also see [52]). Furthermore, although the results of this study suggest that SME leaders are a population with high overall levels of personality functioning, it cannot be ruled out that participants may experience problems in their daily lives. Thus, it is possible that SME leaders may still experience conflicts that cannot be captured by the measurement tools or that are not queried. Therefore, there is a need for management and coaching interventions to make hidden conflicts and needs visible and to address them.

### Implications for leader-member dynamics

As previously stated in relation to potential core dimensions of personality functions, the influence of mental stress on managerial behavior is not only significant at the individual level, but also in terms of the broader leader-member dynamic. Conversely, positive professional relationships serve as an influential protective factor for mental and physical health [53, 54].

This seems in line with prior research which has demonstrated a negative association between depressive and anxiety symptoms in leaders and their leadership behavior, particularly with regard to transformational leadership traits, such as providing followers with individualized support [14]. Thus, mental health is not only a consequence of effective leadership, but also a fundamental element that enables effective leadership to flourish [55].

Furthermore, findings by Diebig et al. [56] indicate that leaders who exhibit high levels of stress reactivity tend to experience challenges in fostering positive and high-quality relationships with their followers. More specifically, it was also found that the individual Leader-Member-Exchange (LMX) relationship quality formed between a leader and each follower within the team (LMX differentiation) is associated with followers’ psychological strain on the individual, yet also on the level of the whole team [10]. A low LMX relationship between followers and their leaders has been linked to elevated stress levels and feelings of disadvantageous treatment compared to other team members. However, this relationship tends to diminish when leaders strive for an equal LMX relationship towards all team members. In light of this, Diebig et al. [56] propose that leaders should strive to enhance their overall LMX level with all employees, with the aim of fostering fair and equitable relationships within teams and reducing interpersonal discrepancies.

Thus, our findings align with those of previous research, which indicate that leaders can only foster positive relationships and demonstrate effective leadership if they possess the requisite psychological capabilities [40] underlining the need to address leader´s mental health appropriately [57]. In the light of this background, systemic approaches targeting the relationship between leaders and followers seem to be a useful source for intervention. For instance, by employing circular, systemic inquiries and shifts in perspective, leaders can discern how their emotional states are shaped by their role within the system and cultivate a more nuanced comprehension of how they can articulate their feelings with greater clarity.

### Implications and interventions for leaders’ mental health

The findings have implications for the development of health promotion strategies and health-related leadership competencies for managers in small and medium-sized enterprises (SMEs). Existing studies underscore the connection between proficient emotional skills and emotional intelligence, which are associated with superior-quality relationships and positive effects in the work context [58–61]. As affect-related and emotion-focused subscales have been demonstrated to be significant in our study with regard to levels of mental distress, it is recommended that leadership training or coaching should explicitly address the mechanisms of emotions and emotion regulation.

Incorporation of these findings may facilitate the formation of a more refined emotional self-perception and improved influence on previously automated inner-psychic dynamics [18]. The results confirm the importance of promoting aspects such as self-esteem, self-management and self-responsibility in training programmes or interventions for SME leaders. Self-reflection, guided by trainers or peers, provides an opportunity to reflect on one’s individual strengths and to receive feedback. Similarly, conducting positive feedback sessions in which colleagues or trainers highlight strengths and special achievements can be seen as helpful. Providing knowledge of mindfulness and meditation techniques can be beneficial for managers to help them deal with stressful situations and focus on essential aspects [62, 63]. CBT-based approaches may be incorporated with the aim of identifying and modifying maladaptive thought patterns and behaviours, thereby enhancing emotional regulation and leadership abilities, facilitating a more accurate identification and comprehension of emotions. In the light of the findings of this study and akin to existing CBT-based interventions for SME leaders (see [64, 65], future interventions could foster SME leaders to observe their emotional reactions to stressful situations and to perceive them in a more differentiated manner. This might foster resilience to criticism and failure, and a stable perception of self-esteem, independently of external successes. Furthermore, the implementation of psychodynamic oriented approaches might enable leaders to delve more profoundly into their unconscious conflicts and earlier relationship experiences, which in turn facilitates a more nuanced understanding of the emotional regulation and personality functions that shape their professional conduct.

The findings of existing studies conducted in a variety of work settings indicate that emotional skills can be effectively trained within the workplace [66–68].

### Limitations

To the best of our knowledge, this study is the first multicenter study to investigate the role of self-assessed personality functioning on perceived stress reactivity (PSR) and symptoms of anxiety and depression among in the group of leaders of small-and medium sized enterprises (SMEs).

In addition to the strengths of this study, some limitations should not be disregarded. The study took place from May to August 2021, amidst the impact of the Corona pandemic in Germany. Therefore, the external factors of pandemic-related supply shortages, home working days, and sickness absence must be acknowledged. These factors created difficulties in recruiting leaders and may have contributed to biased selection of SMEs with enough resources to participate in the study. Also, it should be noted that the study assessed SME leaders in two highly affluent and structurally and economically highly supported regions of southern Germany. Thus, the outcomes may differ in other regions inside and outside Germany.

The objective of our study was to investigate the contribution of SME leaders’ self-rated levels of personality functioning on the subscales self-perception and self-reflection on PSR and symptoms of anxiety and depression. It is to be noted that the cross-sectional design of this study presents certain limitations with regard to the formulation of causal inferences. Nevertheless, due to the underlying theoretical assumptions of the concept of personality functioning, we assume an impact on mental distress using regression models. Further research, especially longitudinal designs, may contribute to a more elaborate investigation on this associations. It is also important to note that additional unassessed variables may have an impact on the statistical modelling between personality functioning and mental distress. Further studies should investigate and incorporate other factors that affect the perceived stress of leaders of small and medium-sized enterprises. Further research initiatives may include additional dimensions of the Operationalized Psychodynamic Diagnosis system (OPD) for a broader understanding of mental distress in SME leaders.

## Conclusion

The aim of this study was to investigate the contributing role of personality functioning (operationalized by the subscales of *self-perception* and *self-regulation*) on mental distress (operationalized by perceived stress reactivity and symptoms of anxiety and depression) among leaders of small-and medium sized enterprises (SMEs).

We were able to show that one facet of the subscale self-perception (i.e. affect differentiation) and three facets of the subscale self-regulation (i.e. affect tolerance, regulation of self-esteem and impulse control) mainly contributed to the explanation of mental distress among SME leaders. The findings of this study underscore the impact of the availability of personal functioning regarding self-perception and self-regulation that are inherent to leaders of SMEs which can in turn impact the experience of both external and internal stressors. We anticipate that the identified facets can enhance our comprehension of how SME leaders undergo their surroundings, regulate their emotions and impulses, and therefore impact their susceptivity towards the experience of stress, anxiety and depression.

Our findings can be extended to the need for more high-quality randomized controlled trials that examine the health of leaders and the precursors of mental health in further depth and offer prevention and personalized healthcare interventions.

## Supporting information

S1 Table Illustration of the correlations between the study variables and Cronbach’s alpha on the diagonal. Strong correlations (≥ .50) are shown in dark grey, moderate correlations (≥ .30) in light grey, and weak correlations (< .30) in white.
(DOCX)
