# Supplementary material for: Personality functioning and mental distress in leaders of small- and medium sized enterprises
Source: PLoS One. 2024 Nov 20;19(11):e0312675. doi: 10.1371/journal.pone.0312675 (PMC11578471; doi:10.1371/journal.pone.0312675)
Supplement: S1 Table — Strong correlations (≥ .50) are shown in dark grey, moderate correlations (≥ .30) in light grey, and weak correlations (< .30) in white. (DOCX) [file pone.0312675.s001.docx]

**Supporting Information**

**S1 Table.** **Illustration of the correlations between the study variables and Cronbach's alpha on the diagonal.** Strong correlations (≥ .50) are shown in dark grey, moderate correlations (≥ .30) in light grey, and weak correlations (< .30) in white*.*

|  | 1. | 2. | 3. | 4. | 5. | 6. | 7. | 8. | 9. | 10. | 11. | 12. | 13. | 14. | 15. | 16. |
| --- | --- | --- | --- | --- | --- | --- | --- | --- | --- | --- | --- | --- | --- | --- | --- | --- |
| 1. OPD-self-perception sum score | **.91** |  |  |  |  |  |  |  |  |  |  |  |  |  |  |  |
| 2. self-reflection | .84*** | **.78** |  |  |  |  |  |  |  |  |  |  |  |  |  |  |
| 3. affect differentiation | .91*** | .63*** | **.89** |  |  |  |  |  |  |  |  |  |  |  |  |  |
| 4. sense of identity | .83*** | .55*** | .66*** | **.81** |  |  |  |  |  |  |  |  |  |  |  |  |
| 5. OPD-self-regulation sum score | .73*** | .55*** | .72*** | .59*** | **.86** |  |  |  |  |  |  |  |  |  |  |  |
| 6. impulse control | .44*** | .37*** | .44*** | .31*** | .77*** | **.76** |  |  |  |  |  |  |  |  |  |  |
| 7. affect tolerance | .71*** | .47*** | .73*** | .61*** | .86*** | .47*** | **.87** |  |  |  |  |  |  |  |  |  |
| 8. regulation of self-esteem | .61*** | .52*** | .56*** | .50*** | .79*** | .37*** | .59*** | .**67** |  |  |  |  |  |  |  |  |
| 9. PSRS sum score | .62*** | .49*** | .61*** | .48*** | .70*** | .47*** | .58*** | .66*** | **.89** |  |  |  |  |  |  |  |
| 10. prolonged reactivity | 54*** | .44*** | .52*** | .41*** | .46*** | .33*** | .40*** | .37*** | .68*** | **.77** |  |  |  |  |  |  |
| 11. reactivity to work overload | .47*** | .32*** | .48*** | .38*** | .57*** | .43*** | .49*** | .46*** | .77*** | .47*** | **.78** |  |  |  |  |  |
| 12. reactivity to social conflicts | .35*** | .22** | .39*** | .29*** | .50*** | .33*** | .41*** | .46*** | .80*** | .43*** | .48*** | **.78** |  |  |  |  |
| 13. reactivity to failure | .46*** | .40** | .40*** | .40*** | .54*** | .32*** | .43*** | .58*** | .75*** | .37*** | .46*** | .60*** | **.67** |  |  |  |
| 14. reactivity to social evaluation | .52*** | .50*** | .50*** | .35*** | .57*** | .33*** | .56*** | .61*** | .76*** | .32*** | .47*** | .50*** | .53*** | **.68** |  |  |
| 15. HADS anxiety | .59*** | .42*** | .61*** | .47*** | .60*** | .35*** | .62*** | .46*** | .63*** | .58*** | .55*** | .47*** | .46*** | .35*** | **.77** |  |
| 16. HADS depression | .47*** | .35*** | .46*** | .41*** | .47*** | .32*** | .40*** | .44*** | .50*** | .51*** | .39*** | .36*** | .39*** | .26*** | .56*** | **.80** |

*Note.* OPD = Operationalized Psychodynamic Diagnosis system; PSRS = Perceived Stress Reactivity Scale, HADS = Hospital Anxiety and Depression Scale

**p*<.05, ***p*<.01, ****p*<.001
